# Loss of Knee Flexion and Femoral Rollback of the Medial-Pivot and Posterior-Stabilized Total Knee Arthroplasty During Early-Stance of Walking in Chinese Patients

**DOI:** 10.3389/fbioe.2021.675093

**Published:** 2021-06-24

**Authors:** Jiaqi Tan, Diyang Zou, Xianlong Zhang, Nan Zheng, Yuqi Pan, Zhi Ling, Tsung-Yuan Tsai, Yunsu Chen

**Affiliations:** ^1^Department of Orthopedic Surgery, Shanghai Sixth People’s Hospital, Shanghai Jiao Tong University, Shanghai, China; ^2^School of Biomedical Engineering & Med-X Research Institute, Shanghai Jiao Tong University, Engineering Research Center of Digital Medicine and Clinical Translation, Ministry of Education, Department of Orthopaedic Surgery, Shanghai Key Laboratory of Orthopaedic Implants and Clinical Translational R&D Center of 3D Printing Technology, Shanghai Ninth People’s Hospital, Shanghai Jiao Tong University School of Medicine, Shanghai, China

**Keywords:** total knee arthroplasty, posterior-stabilized, medial-pivot, gait, *in vivo* kinematics, patient satisfaction

## Abstract

**Background** The medial-pivot (MP) prosthesis was developed to produce more physiological postoperative knee kinematics and better patient satisfaction than traditional prostheses, but outcomes are inconsistent in different studies of Caucasian patients. This study aimed to investigate the postoperative patient satisfaction and *in vivo* knee kinematics of the MP and posterior-stabilized (PS) prosthesis during gait activity in Chinese patients.

**Methods** A retrospective analysis of 12 patients was received for this study in each MP group and PS group. Patient-reported satisfaction level and Forgotten Joint Score (FJS) were evaluated with questionnaires. A dual fluoroscopic imaging system was used to investigate *in vivo* knee kinematics of MP and PS total knee arthroplasty (TKA) during treadmill walking at a speed of 0.4 m/s.

**Results** Comparable promising patient satisfaction and overall FJS (MP 60.7 ± 15.35 vs. PS 51.3 ± 17.62, *p* = 0.174) were found between the MP and PS groups. Peak flexion appeared at around 70% of gait cycle with values of 52.4 ± 7.4° for MP and 50.1 ± 3.6° for PS groups (no difference). Both groups maintained a stable position at the stance phase and began to translated anteriorly at toe-off with an amount of 4.5 ± 2.3 mm in the MP and 6.6 ± 2.7 mm in the PS (*p* = 0.08) group until late swing. The range of this external rotation motion was 5.9 ± 4.8 and 6.2 ± 4.1° (*p* = 0.79) for the MP and PS, respectively.

**Conclusion** A similar knee kinematics pattern characterized by a loss of early-stance knee flexion and femoral rollback during walking was observed in the MP and PS TKAs. Our study confirmed similar effectiveness of MP TKA compared to PS TKA in Chinese patients, while the change of knee kinematics of both implants during slow walking should be noted.

## Introduction

Since its introduction in the 1950s, total knee arthroplasty (TKA) has been the most effective and successful treatment for late-stage knee arthritis (Nguyen et al., 2015). However, about 20% of TKA patients reported a lack of satisfaction (Bourne et al., 2009). Patients usually complain about the knee’s instability and an abnormal feeling of the knee joint during daily activities (Nam et al., 2014). One reason is the alteration of the knee articular surfaces resulting in changes of the knee kinematics (Dunbar et al., 2013).

Posterior-stabilized (PS) TKA and posterior cruciate ligament (PCL) retaining (CR) TKA are the two most commonly used knee prostheses today. Studies have shown the PS TKA is manifested with paradoxical anterior femoral sliding and abnormal tibiofemoral axial rotation during walking and knee flexion activities (Schmidt et al., 2003; Banks and Hodge, 2004; Victor et al., 2010; Bae et al., 2016). This phenomenon is also evident in CR knees (Dimitriou et al., 2016; Vince, 2016; Zeller et al., 2017). These abnormalities of the knee kinematics may affect patient satisfaction and the implant’s durability. The medial-pivot (MP) prosthesis is designed to mimic normal knee kinematics. The MP prosthesis includes a ball and socket articulation on the medial side with more significant restriction and relatively unconstrained mobility on the lateral side. The tibial insert’s particular geometries provide anteroposterior translational stability and allow the knee to rotate in a MP pattern. However, clinical outcomes of MP TKA are inconsistent in the literature. Numerous studies reported satisfactory clinical results of MP TKA (Hossain et al., 2011; Youm et al., 2014; Steinbruck et al., 2016; Gray et al., 2020; Jones et al., 2020) and patient preference over PS and other prostheses (Schmidt et al., 2003; Pritchett, 2011). On the contrary, other authors reported that the MP design was not superior to other designs in patient-reported outcomes and knee kinematics (Chinzei et al., 2014; Papagiannis et al., 2016; Choi et al., 2017; Kim et al., 2017; Jones et al., 2020; Nisar et al., 2020). More evidence is needed to determine the effectiveness and design specialty of the MP prosthesis.

Most studies of MP TKA are conducted in Caucasian patients. Data regarding Chinese MP TKA patients are limited and conflicting. Lin et al. (2020) reported a similar short-term patient satisfaction rate of MP TKA and PS TKA (87.38 vs. 89.89%, *p* = 0.75) in Chinese patients. Also, Yuan et al. (2020) revealed comparable midterm patient-reported functional outcomes between MP and PS TKA. In contrast, in a 6.5-year follow-up of 572 patients, Shi et al. (2020) reported no significant differences amount clinical outcomes, range of motion, and postoperative complications between two types of protheses. Compared to patient-reported outcomes, *in vivo* kinematics measurement is a more objective and accurate method to investigate the postoperative behavior of different artificial knee designs. Accurate quantitative data are required to evaluate the differences between the MP and PS TKA in Chinese patients. To the author’s best knowledge, there is no data available regarding *in vivo* knee kinematics in Chinese MP TKA patients during daily activities, such as walking. Some studies have investigated knee kinematics during gait activity in Caucasian patients (Papagiannis et al., 2016; Benjamin et al., 2018; Esposito et al., 2020; Miura et al., 2020), reporting better mimicking of normal knee kinematics of MP TKA than other designs. Nevertheless, Chinese patients are manifested with more severity of osteoarthritis and walking disabilities than Caucasian patients before surgery (Zhang et al., 2001; Kang et al., 2009; Lin et al., 2010; Liu et al., 2017), which may restrict their function restoration after TKA surgery, such as adopting a slow walking speed. Walking speed has been proved influential to knee kinematics after total knee replacement (Tarnita et al., 2020). Most modern total knee prostheses, including the MP prosthesis, are designed based on Caucasian anthropometries. These factors render it foggy whether Chinese patients benefit from this kinematics-friendly MP design for TKA.

The objective of this study is to investigate the effectiveness of the MP TKA in Chinese patients with comparison to the PS TKA by measuring (1) postoperative patient satisfaction level and Forgotten Joint Score (FJS) and (2) postoperative *in vivo* knee kinematics during gait. We hypothesize that different knee kinematics patterns during walking and patients reported outcomes were observed in MP and PS TKAs.

## Materials and Methods

### Study Design

**FIGURE 1:**
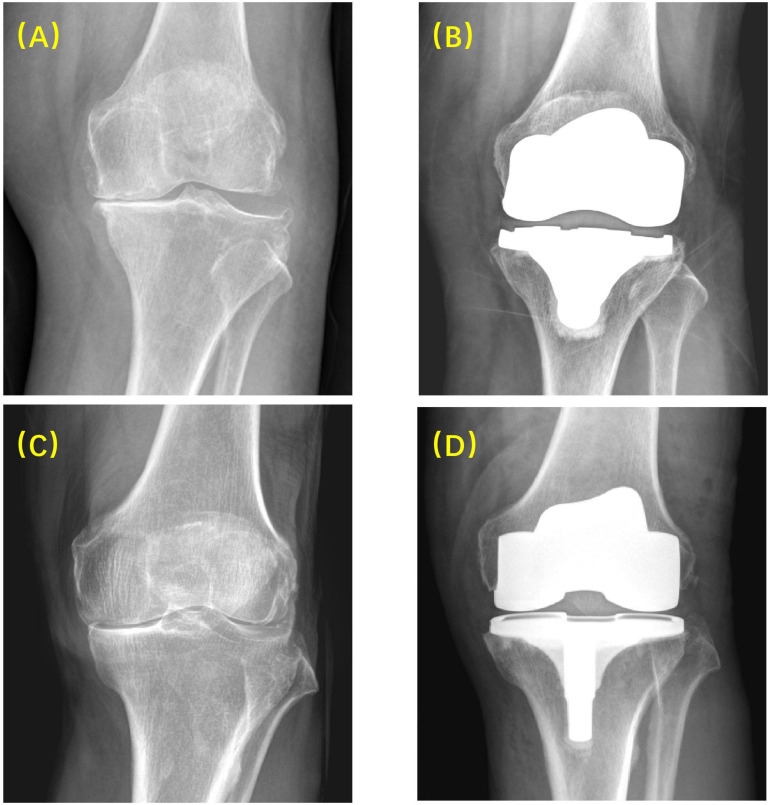
Radiographic images before and after surgery using MP and PS prostheses. **(A)** Before MP TKA; **(B)** after MP TKA; **(C)** before PS TKA; **(D)** after PS TKA.

In this study, 12 patients who had undergone a PS (Genesis II, Smith and Nephew, Memphis, TN, United States) knee replacement and 12 patients who had undergone an MP (Evolution, MicroPort Orthopedics, Arlington, TN, United States) knee replacement during 2016–2019 at our institution were recruited (Figure 1 and Table 1). Inclusion criteria were late-stage primary osteoarthritis with varus or neutral deformity, passive flexion more than 90°, and good collateral stability. Priori power analysis (G^∗^Power version 3.1; Franz Faul, Universität Kiel, Germany) was performed to estimate the minimum sample size, with α = 0.05 and effect size = 1.9 which calculated according to the difference of antero-posterior translation in lateral tibial component (Hosseini Nasab et al., 2019). Each group’s number of 7 is enough to evaluate the difference between the two groups with power of 0.96. Patients diagnosed with rheumatoid arthritis, posttraumatic arthritis, or osteoarthritis with valgus deformity, or those who had undergone a knee replacement before, were excluded. All patients were informed of the nature of the study and provided informed consent. The study was approved by the Ethics Committee at our institution (Protocol Code: YS-2018-124; Date of Approval: June 12, 2018).

**TABLE 1:** Patient demographics and postoperative time.

**Variables**	**MP^1^**	**PS^2^**	***p*-Value**
Gender (F:M)	9:3	9:3	–
Age, *y*	70.8 (±3.9) range 65–76	67.7 (±4.9) range 60–74	0.126
Height, *cm*	159.5 (±7.4)	158.6 (±7.5)	0.783
Weight, *kg*	69.8 (±7.9)	68.0 (±11.4)	0.703
BMI, *kg/m^2^*	27.4 (±2.6)	26.9 (±2.9)	0.668
Postoperative Time, *m*	25.2 (±2.5)	26.0 (±2.3)	0.464
Preoperative HKA	173.4 (±5.2)	172.9 (±4.3)	0.575
Postoperative HKA	178.4 (±1.1)	178.1 (±1.1)	0.265

*^1^MP, medial pivot. ^2^PS, posterior-stabilized.*

All surgeries were performed by one senior surgeon, following a standard surgical technique in both MP and PS groups, including a midline incision, parapatellar exposure, intramedullary distal femoral resection, and extramedullary tibial resection. The patella was not resurfaced. A mechanical alignment method was employed (Rivière et al., 2017; Dehl et al., 2018). All patients received standard postoperative care and were asked to follow an identical rehabilitation protocol.

### Patient Satisfaction Level

The patient satisfaction level was determined by asking patients four questions: (1) Are you satisfied with the TKA surgery? (2) Are you satisfied with the TKA surgery relieving your pain? (3) Are you satisfied with the TKA surgery improving your ability of doing housework? (4) Are you satisfied with the TKA surgery improving your ability of doing recreational activities? For each question, a four-level satisfaction rating (very satisfied, satisfied, dissatisfied, or very dissatisfied) was included. The patient number and percentage of each satisfaction level were calculated and compared between the two groups. Patient satisfaction level rating questionnaire followed Lin et al. (2020) as reference.

### Forgotten Joint Score

The FJS (Behrend et al., 2012) is a validated method of evaluating patient satisfaction after TKA surgery and particularly focuses on patients’ proprioception of the operated knee. The FJS consists of 12 questions, and for each question, the patients were asked to answer “Never,” “Almost never,” “Seldom,” “Sometimes,” or “Mostly,” with a score from 0 to 4, respectively. For each patient, the total score was converted into a 100 scaled final score with the following formula: Final score = 100 − [(*A*/*B*) × 25]. *A* was the total score of each patient, and *B* was the number of questions. Higher final scores indicate better outcomes.

### *In vivo* Kinematics Measurement

All patients received a computer tomography (CT) scan (Discovery CT750 HD, GE MEDICAL SYSTEMS, United States, 120 kVp, image resolution 512 × 512 pixels, voxel size 0.86 mm × 0.86 mm × 0.63 mm) from the femoral head to the ankles postoperatively. Images were imported into medical image software (Amira 6.7.0, Thermo Fisher Scientific, Hillsboro, OR, United States) to reconstruct the three-dimensional (3D) models of both knees, including bony surfaces of the femur and tibia. The 3D models of the MP and PS TKA prostheses were obtained from the manufacturers or laser scanning (Sami et al., 2020).

The patients were instructed to walk on a treadmill under a dual fluoroscopic imaging system (BV Pulsera, Philips, Andover, MA, United States, image resolution 1024 × 1024 pixels, dynamic image frequency 30 Hz). After 15 min of training, a speed of 0.4 m/s was reported comfortable by patients. The fluoroscopic images, 3D prostheses models, and 3D bone models were imported into a customized software in MATLAB (MATLAB 2020a, MathWorks, Natick, MA, United States). The 3D pose of the TKA component was determined with a previously published protocol (Jeffrey Bingham and Li, 2006) by best matching the silhouette of the prosthesis 3D models to that of the dynamic fluoroscopic images.

**FIGURE 2:**
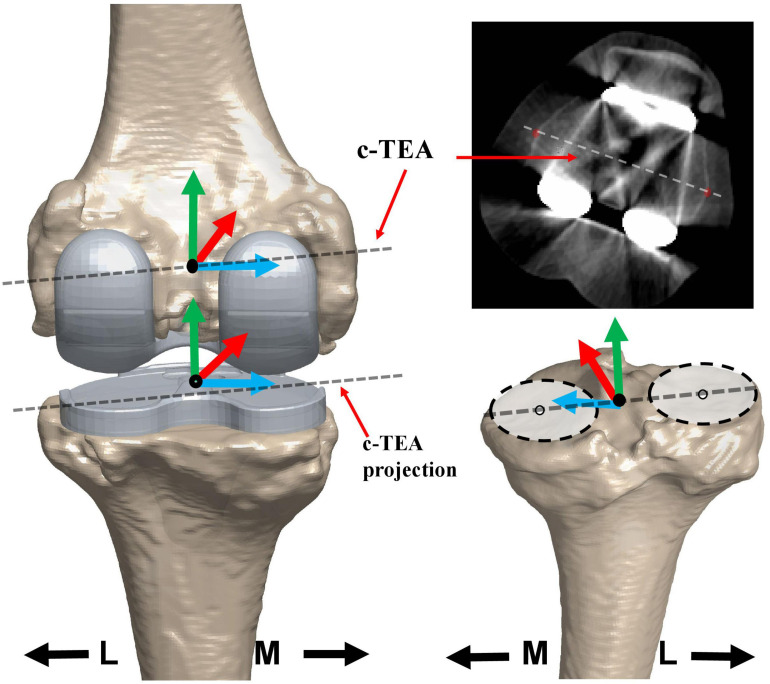
The local coordinate system of the femur and the tibia. The coordinate systems of the femur and tibia are based on the 3D knee model of MP TKA patients reconstructed from computer tomography (CT) images. The dotted line on the 3D model and in the CT image of the femoral prostheses was the clinical trans-epicondylar axis (TEA) of the femur. The dotted line on the tibial prosthesis was the projection of the clinical trans-epicondylar axis (TEA). The dotted line on the non-operated tibial plateau was the line connecting the two best-fitted circles. The black solid point indicated the center of the femur or tibia. The red, green, and blue lines showed the anterior/posterior (AP), proximal/distal (PD), and medial/lateral (ML) axes, respectively.

The local coordinate system of the femur and tibia on the operated side was established separately. For the femoral, the anatomical coordinate system was set directly on the operated side based on 3D models (Kozanek et al., 2009), as the bony landmarks required were not removed by bone resection during TKA surgery. The midpoint of the clinical transepicondylar axis (c-TEA) was defined as the femoral center. The line between the center of the femoral head and the femoral center was the long axis (proximal/distal axis: P/D axis). A line through the femoral center and perpendicular to the plane determined by the long axis and c-TEA line was made to be the anterior/posterior (A/P) axis. The cross product of the A/P axis and the long axis was set as the medial/lateral (M/L) axis (Figure 2). The tibial anatomical coordinate system was determined first on the non-operated side and subsequently flipped to the operated side using a previously published and validated 3D mirroring technique (Tsai et al., 2014), through minimizing the surface-to-surface registration errors between the operated tibia and mirrored non-operated tibia. Two best-fit circles were created for non-operated medial and lateral tibial plateaus separately. The midpoint of the line connecting the two circle centers was defined as the tibial center (Figure 2). The proximal tibial long axis (P/D axis) was defined as the line connecting the tibial center and the distal tibial center. The line through the tibial center and perpendicular to the plane constituted by the two tibial plateau circle centers, and the distal tibial center was set as the tibial A/P axis. The cross product of the tibial A/P and long axis was defined as the tibial M/L axis of the tibia. The c-TEA was projected to the tibia to represent anteroposterior translation of medial and lateral femoral condyle (Figure 2).

The knee kinematic parameters, including translations and rotation angle, were calculated according to the recommendation of the International Society of Biomechanics (ISB) (Wu et al., 2002). The positive value of rotation was defined as the internal rotation of the tibia around the P/D axis of the femur. The positive value of translation was defined as the anterior, medial, and proximal movement of the femur with respect to the tibia along the A/P, M/L, and P/D axes. The root means square errors of rotational and translational parameters had been validated for less than 1° and 1 mm previously (Hanson et al., 2006).

### Statistical Analysis

Patient characteristics were displayed as means ± standard deviations and were analyzed with a *t*-test. Gender was presented as female:male and satisfaction as patient number (percentage). The Fisher exact probability test was used for gender and satisfaction analysis. FJS was analyzed with a *t*-test. A Mann–Whitney U test was used to compare the difference of *in vivo* kinematics between MP TKAs and PS TKAs. A significant difference level was set as *p* < 0.05. The statistical analyses were conducted using MATLAB (MATLAB 2020a, MathWorks, Natick, MA, United States).

## Results

### Patient Satisfaction

To investigate patient satisfaction in each group, we collected a survey composed of four questions from patients. As to pain relief (question 2) of the TKA surgery, the number of satisfied patients in the MP group was larger than the PS group (MP 12 vs. 10 PS) (Table 2). In addition, as to general impression and improvement of the ability to do housework and recreational activities (questions 1, 3, and 4), the same number of satisfied patients were found in both groups, while the PS group had more “very satisfied” patients than MP group for each of the three questions (PS 10 vs. 7 MP, PS 10 vs. 8 MP, and PS 9 vs. 7 MP, respectively) (Table 2). Nevertheless, all the differences were not statistically significant, indicating no superiority of MP TKA over PS TKA in terms of patient satisfaction.

**TABLE 2:** Patient Satisfaction Questionnaire.

**Satisfaction level**	**MP^1^**	**PS^2^**	***p-*Value**
1. Are you satisfied with the TKA surgery?			
Very satisfied	7(0.58)	10(0.83)	0.317
Satisfied	4(0.33)	1(0.08)	0.371
Dissatisfied	1(0.08)	1(0.08)	–
Very dissatisfied	0(0)	0(0)	–
2. Are you satisfied with the TKA surgery relieving your pain?			
Very satisfied	10(0.83)	10(0.83)	–
Satisfied	2(0.17)	0(0)	0.478
Dissatisfied	0(0)	2(0.17)	0.777
Very dissatisfied	0(0)	0(0)	–
3. Are you satisfied with the TKA surgery improving your ability of doing housework?			
Very satisfied	8(0.67)	10(0.83)	0.640
Satisfied	3(0.25)	1(0.08)	0.590
Dissatisfied	1(0.08)	1(0.08)	–
Very dissatisfied	0(0)	0(0)	–
4. Are you satisfied with the TKA surgery improving your ability of doing recreational activities?			
Very satisfied	7(0.58)	9(0.757)	0.667
Satisfied	4(0.33)	2(0.17)	0.640
Dissatisfied	1(0.08)	1(0.08)	–
Very dissatisfied	0(0)	0(0)	–

*^1^MP, medial pivot. ^2^PS, posterior-stabilized. Data were presented as patient number (percentage).*

### Forgotten Joint Score

In our study, none of the patients reported having a sporting habit. Therefore, question 12 was eliminated from the FJS questionnaire for all patients, and the final score was calculated based on the left 11 questions. A lower score for individual question and a higher final score indicated better outcomes. In the overall FJS, we found no statistical differences between MP (60.7 ± 15.35) and PS (51.3 ± 17.62) TKA groups (*p* = 0.174; Table 3). However, there was a statistically significant difference between the two groups for question 5 (MP 0.8 ± 0.83 vs. 1.8 ± .94 PS; *p* = 0.026) and question 8 (MP 2.3 ± 1.15 vs. 3.3 ± 0.75 PS; *p* = 0.042), when patients traveled in a car and rose from a low position, respectively (Table 3). In both questions, MP TKA was better than PS TKA.

**TABLE 3:** Forgotten Joint Score.

**Question: Are you aware of your artificial knee joint when**	**MP^1^**	**PS^2^**	***p-*Value**
1. In bed at night?	0.5 (±0.67)	1.0 (±1.41)	0.324
2. Sitting in chair >1 h?	1.5 (±1.17)	2.0 (±1.48)	0.275
3. Walking >15 min?	1.4 (±0.99)	1.8 (±0.97)	0.474
4. Taking a bath/shower?	1.2 (±1.19)	1.7 (±1.23)	0.275
5. Traveling in a car?*	0.8 (±0.83)	1.8 (±0.94)	0.026*
6. Climbing stairs?	2.3 (±1.14)	3.1 (±0.90)	0.096
7. Walking on uneven ground?	2.6 (±0.90)	2.2 (±1.03)	0.339
8. Rising from a low sitting position?*	2.3 (±1.15)	3.3 (±0.75)	0.042*
9. Standing for a long period of time?	1.0 (±0.85)	1.8 (±1.27)	0.075
10. Doing housework?	1.6 (±0.79)	1.3 (±1.07)	0.555
11. Taking a walk/hike?	2.3 (±0.87)	1.5 (±1.17)	0.069
12. Doing your favorite sport?^3^	–	–	–
Total score	60.7 (±15.35)	51.3 (±17.62)	0.174

^1^MP, medial pivot. ^2^PS, posterior-stabilized. ^3^None of our patients in this study reported having a sport habit. *p < 0.05 was statistically significant.

### The Tibiofemoral Kinematics

**FIGURE 3:**
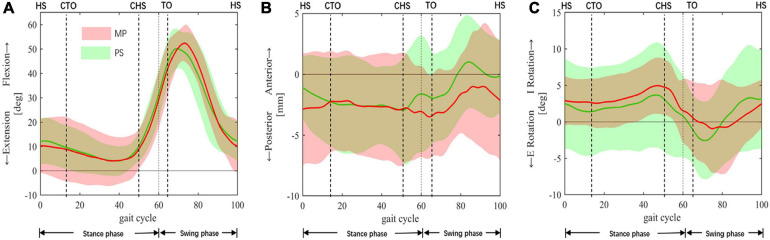
*In vivo* kinematics of TKA knees. *In vivo* kinematics of the medial pivot (MP: red color) and the posterior-stabilized (PS: green color) TKAs during a complete gait cycle. **(A)** Knee Flexion/Extension. **(B)** Anterior/posterior femoral translation was relative to the tibia. **(C)** Internal/external tibial rotation was relative to the femur. The means and standard deviations of flexion+/extension–, anterior+/posterior– translations, internal+/external– rotation (I/E rotation), were represented by solid lines and corresponding shaded area. The dashed lines at 60% cut the gait cycle into the stance phase and swing phase. Gait events include HS, heel-strike; CTO, contralateral toe-off; CHS, contralateral heel-strike; TO, toe-off.

In both groups, the knee only flexed once in swing phase, with a peak flexion at around 70% gait cycle and values of 52.4 ± 7.4° for MP and 50.1 ± 3.6° for PS (Figure 3A), and no difference of statistical significance was found (*p* = 0.47). In the early stance phase, the femur of MP TKA translated anteriorly on the tibia, while for PS TKA, the femur rolled back (Figure 3B). Thereafter, both femurs maintained their positions on the tibia. In late stance right after contralateral heel-strike, the MP femur moved posteriorly, and the PS femur moved anteriorly. After entering the swing phase, femurs in both groups began to translated anteriorly at toe-off with an amount of 4.5 ± 2.3 mm in MP and 6.6 ± 2.7 mm in PS (*p* = 0.08) until late swing. Then both femurs began to translate posteriorly as the knee flexed. No statistical difference was found in femoral anteroposterior translations between MP and PS groups. In the most period of stance phase, the MP tibial and PS tibia showed a consistent slow internal rotation until closely before contralateral heel-strike, they both turned into a motion of sharp external rotation as the knee began to flex and the femur began to translate anteriorly (Figure 3). The range of this external rotation motion was 5.9 ± 4.8 and 6.2 ± 4.1° (*p* = 0.79) for MP and PS, respectively (Figure 3C). The external rotation of both tibiae reached approximately at the same time of flexion peak, and it is followed by internal tibial rotation as the knee extended (Figures 3A,C).

### Anteroposterior Translation of the Femoral Condyles

**FIGURE 4:**
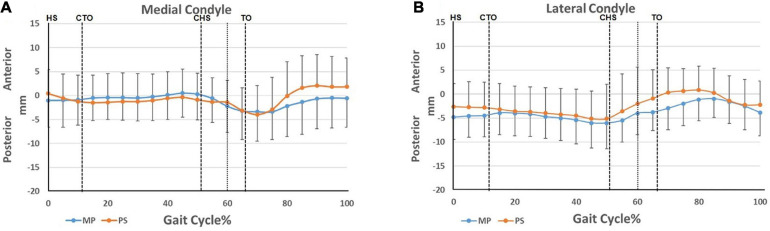
Condylar motion of the medial and lateral condyles relative to the tibia. **(A)** Anterior/posterior translation of the medial femoral condyles of the MP and PS TKA; **(B)** anterior/posterior translation of the lateral femoral condyles of the MP and PS TKA. Blue lines: MP TKA. Red lines: PS TKA. The means and standard deviations of translations were represented by solid lines and short vertical bars. The dashed lines at 60% cut the gait cycle into stance phase and swing phase. Gait events include: HS, heel-strike; CTO, contralateral toe-off; CHS, contralateral heel-strike; TO, toe-off.

In the anterior/posterior direction, from heel-strike to contralateral heel-strike, the medial condyles in both groups generally maintained their position with slight translation relative to the tibia (Figure 4A). The lateral condyles moved posteriorly (Figure 4A), displaying a medial pivoting and contributing to internal tibial rotation (Figure 3C). Following that, as the knee began to flex from the pre-swing phase to flexion peak, the medial condyles of both TKA knees moved posteriorly (MP 3.9 ± 5.5 vs. 3.6 ± 6.0 mm, *p* = 0.71). In contrast, the lateral condyles in both groups moved anteriorly (MP 8.9 ± 9.2 vs. 4.0 ± 4.7 mm, *p* = 0.89) (Figures 4A,B). This condylar motion contributed to the external tibial rotation between contralateral heel-strike to flexion peak (Figure 3C). Then, with knee extension, the medial condyles in both TKA groups began to translate anteriorly, and the lateral condyles kept moving anteriorly, which accompanied the femoral anterior sliding during this phase as the knee flexed. Subsequently, after 80% of the gait cycle, the medial condyles maintained their anteroposterior position while the lateral condyles moved posteriorly (Figures 4A,B). This constituted the femoral roll-back and internal tibial rotation (Figures 3B,C) and showed a medial pivoting motion.

## Discussion

In this study, we measured postoperative satisfaction and the knee kinematics of MP and PS TKAs in Chinese patients during walking. Patient satisfaction and overall FJSs were similar between the MP and the PS groups, showing good effectiveness of the MP prosthesis in Chinese, compared to the PS prosthesis on a small sample size. *In vivo* knee kinematics during gait revealed the absence of the knee flexion and femoral rollback in the early stance phase, which may affect the effectiveness of the knee extensor mechanism and cause potential instability in Chinese patients during different activities.

Postoperative patient satisfaction was evaluated with patient satisfaction rate and FJSs. Lin et al. (2020) reported similar short-term patient satisfaction rate of MP TKA and PS TKA (87.38 vs. 89.89%, *p* = 0.75) in Chinese patients. In our study, the patients reported desirable satisfaction rates both in MP (92% for each of four questions) and PS (92% for question 1, 3, and 4; 83% for question 2) groups (Table 2). This corroborated with Lin et al. (2020) and indicated the excellent effectiveness of MP TKA in Chinese patients. Samy et al. (2018) reported a better overall FJS score of MP than PS (MP 59.7 ± 31.7 vs. 44.8 ± 28.5 PS, *p* = 0.007). In our study, no statistically significant difference was found between the MP and PS groups (MP 60.7 ± 15.4 vs. 51.3 ± 17.6 PS, *p* = 0.007) (Table 3). The disagreement may result from the better FJS score in our PS patients than those in Samy et al.’s (2018) study. Since pain is the most significant factor influencing the quality of life in Chinese knee osteoarthritis patients (Pang et al., 2015), pain relief after surgery may count as the main contributor to a high FJS in both groups instead of function restoration. However, our results corroborated with theirs in terms of the question “Are you aware of your artificial knee joint when you rise from a low position?” In our study, the MP TKA patients reported better FJS scores than PS TKA when traveling in a car. It is expected that the MP TKA may produce better patient satisfaction and FJS in more functionally active patients.

For the first time, we reported the knee kinematics of MP and PS TKA of Chinese patients in this study with the anatomical coordinate system and femoral transepicondylar axis. Gray et al. (2020) reported similar peak flexion angle at mid-swing in the MP and the PS TKA (MP 52.5 vs. 54.8° PS, *p* = 0.64), which was less than healthy knees [70.7° by Gray et al. (2019) and 65.6° by Postolka et al. (2020)]. In our study, the two TKA groups showed similar peak flexion angles (MP 52.4 ± 7.4 vs. 50.1 ± 3.6 PS, *p* = 0.47) and were consistent with previous studies (Yoshida et al., 2012; Arauz et al., 2018; Grieco et al., 2018; Gray et al., 2020). To note, different from TKA knees in other studies of Caucasian patients (Hanson et al., 2006; Gray et al., 2020), no flexion peak in the early-stance phase was found in our patients (Figure 3A). It was accompanied by the absence of femoral rollback in early-stance (Figure 3B). The same phenomenon was observed in another study involving Chinese TKA patients (Zeng et al., 2020). The altered kinematics can be caused by the relatively low walking speed adopted by Chinese patients. The poor athletic ability could be due to a lack of daily activity resulting from function restrictions by severe preoperative osteoarthritis and walking disabilities (Zhang et al., 2001). During normal walking, knee flexion and femoral rollback in early-stance increase the quadriceps’ lever-arm and stabilize the body during weight acceptance (Draganich et al., 2002). The absence of this flexion and femoral rollback is a clinical concern since it could reduce the lever-arm of the quadriceps and comprise knee extensor’s effectiveness, causing feebleness and instability during walking (D’Lima et al., 2001). Due to poor preoperative function, patients with severe preoperative deformities and walking disabilities are more likely to adopt a low walking speed after surgery. Consequently, these patients are more vulnerable to impairment of the knee extensor-mechanism, which could be induced by using an undersized femoral component or posterior positioning of the femoral component (Yehyawi et al., 2007; Antinolfi et al., 2020). For these patients, proper size and sagittal positioning of the femoral component is critical, and surgeons are suggested to take cautions.

In healthy knees, the tibia externally rotated at late swing as the knee extended due to screw-home mechanism of the knee (Kim et al., 2015; Gray et al., 2019). In our study, we found that the tibia in both groups internally rotated (Figure 3C). The difference may be due to the resection of the anterior cruciate ligament, leading to altered knee kinematics and a loss of screw-home motion (Ng et al., 2013). At the same time, our results revealed that medial pivoting only occurred during early- and mid-stance and late swing (Figures 4A,B) in both groups. This could be the consequence of altered articulating surface congruency of the TKA knees compared to healthy knees. Also, a small range of motion during gait activity and low walking speed may weaken the benefit of the particular design of the MP implant. The medial pivoting motion is expected to be more prevalent in more active patients during activities of a more extensive range of motion, as reported by other investigators (Miyazaki et al., 2011; Esposito et al., 2020).

This study has several limitations. First, the study was conducted during treadmill walking instead of overground walking. Investigators have reported that the knee kinematics of treadmill gait could be different from that of overground gait (Riley et al., 2007; Batlkham et al., 2014). It is restrained by our unmovable fluoroscopies, while our patients received adequate training and practice to produce a stable walking pattern. Another limitation is that only gait is involved in a relatively small range of motion, and the influence of different TKA designs on the kinematics of the knee during gait may not be found. Nevertheless, walking is the most fundamental daily activity, and the knee kinematics data during gait is essential to evaluate the *in vivo* functioning of TKA implants in patients. Further study will be needed to evaluate the kinematic differences under different functional activity such as lunge and sit-to-stand. Besides, the sample size in our study is relatively small. As for patient’s satisfaction result, long-term follow-up and study with larger sample size is required for long-term effects. As for the effects in the knee kinematics, a larger sample size can increase the validity of the study. The subtle differences may be found in anterior-posterior translation and internal-external rotation during the swing phase between the two groups. However, the priori power analysis showed the minimum of each group size was 7. Also, similar sample sizes were used in other studies using the same method (Key et al., 2019; Schutz et al., 2019; Postolka et al., 2020).

## Conclusion

In conclusion, our study showed similar gait kinematics and efficiency between the MP TKA and PS TKA in Chinese patients. However, attention should be drawn to the loss of knee flexion and femoral rollback in early-stance in both TKA patients at low-speed walking, which may cause knee instability during daily walking. The function of kinematics-friendly MP design is not inferior to the traditional PS design in gait. As severe preoperative deformities and walking disabilities are highly prevalent in Chinese patients (Liu et al., 2017, 2018), surgeons need to make prudent decisions on optimal surgical management and proper prosthesis selection.

## Data Availability Statement

The raw data supporting the conclusions of this article will be made available by the authors, without undue reservation.

## Ethics Statement

The studies involving human participants were reviewed and approved by the Ethics Committee of Shanghai Sixth People’s Hospital. The patients/participants provided their written informed consent to participate in this study.

## Author Contributions

JT and DZ made substantial contributions to conception and design, acquisition and analysis, and interpretation of the data, involved in drafting the manuscript, and given final approval of the version to be published. XZ, NZ, and YP each partially helped in the acquisition and all given final approval of the version to be published. ZL helped in some data processing and revised the manuscript. T-YT and YC made contributions to conception and design, involved in revising it critically for important intellectual content, and given final approval of the version to be published. All authors contributed to the article and approved the submitted version.

## Conflict of Interest

T-YT received funding from MicroPort Orthopaedics. The funders had no role in the design of the study; in the collection, analyses, or interpretation of data; in the writing of the manuscript, or in the decision to publish the results. The remaining authors declare that the research was conducted in the absence of any commercial or financial relationships that could be construed as a potential conflict of interest.
